# Colour discrimination thresholds in type 1 Bipolar Disorder: a pilot study

**DOI:** 10.1038/s41598-017-16752-0

**Published:** 2017-11-27

**Authors:** Thiago Monteiro Paiva Fernandes, Suellen Marinho Andrade, Michael Jackson Oliveira de Andrade, Renata Maria Toscano Barreto Lyra Nogueira, Natanael Antonio Santos

**Affiliations:** 1 Cognitive Neuroscience and Behaviour Program, Federal University of Paraiba, Joao Pessoa, Brazil; 2 Perception, Neuroscience and Behaviour Laboratory, Federal University of Paraiba, Joao Pessoa, Brazil; 3 Department of Psychology, Federal University of Pernambuco, Pernambuco, Brazil

## Abstract

Although some studies have reported perceptual changes in psychosis, no definitive conclusions have been drawn about visual disturbances that are related to bipolar disorder (BPD). The purpose of the present study was to evaluate colour vision in BPD patients. Data were recorded from 24 participants: healthy control group (*n* = 12) and type 1 BPD group (*n* = 12). The participants were 20–45 years old and they were free from neurological disorders and identifiable ocular disease and had normal or corrected-to-normal visual acuity. Colour discrimination was evaluated using the Lanthony D-15d, Trivector and Ellipse tests, using a psychophysical forced-choice method. The relationship of visual measures to mood state and cognitive function was also investigated. The results showed that BPD patients had higher colour discrimination thresholds in the D15d (*p* < 0.001), Trivector (*p* < 0.001) and Ellipse (*p* < 0.01) tests compared with healthy controls. Linear regression analysis showed that mood state was related to colour discrimination. BPD individuals were not impaired in cognitive tasks. The present study provided new evidence of potential links between type 1 BPD and visual processing impairments. This research suggests a new direction for studies and the need for research in this field of study.

## Introduction

Bipolar disorder (BPD) is a chronic condition with complex pathophysiology that sometimes leads to inefficient diagnosis due to comorbidities or symptoms similar to other diseases^1^. Studies have pointed out that the diversity of symptoms in patients with BPD may lead to a misdiagnosis of major depressive disorder, with inappropriate treatment and perhaps worsening the symptoms or prognosis^1,2^. In this regard, given the nature of the problem, the diagnosis for bipolar needs to be performed carefully.

BPD can be divided into subtypes, generally classified as type 1, type 2, and cyclothymia. Type 1 BPD is characterised by manic or mixed episodes, while type 2 BPD are marked by recurrent episodes of depression and less severe or briefer hypomanic episodes^1^. Depressive symptoms, which are common in the symptomatology of BPD, may be of similar severity in both types 1 and 2; however, type 1 BPD is characterised by repeated manic episodes. In addition, cyclothymic is characterised by hypomania and subthreshold depression^2^.

Colour vision (CV) processing begins in the retina through cone activation and neurotransmitter release^3^. At least three basic characteristics define a colour: hue (i.e., the wavelength that is absorbed by cones), saturation (i.e., colour purity), and brightness (i.e., the number of photons that reach the retina)^4,5^. Colours are processed through luminance channels and two opponent channels for red-green and blue-yellow^6^.

Colour vision-related losses can be either acquired or congenital^7^. Acquired CV loss can result from optical, neural, or systemic disease^8^. Conditions that affect the nervous system, such as BPD, may also affect CV^9–11^, ranging from ophthalmic diseases to pathology of the visual cortex. Bipolar disorder is a complex and heterogeneous disorder that affects the nervous system as a whole^12,13^. Imaging studies have reported a reduction and thinning of fibres in visual processing areas^14,15^. Yet, BPD patients have cortical thinning, regardless of gender or medication^15^. Cortical thinning in the occipital cortex can affect visual processing, suggesting that BPD may be related to impairments in visual processing.

Visual hallucinations in type 1 BPD patients may be associated with biological markers that may be useful for the prognosis and understanding of disturbances of visual perception. Psychiatric disorders that have similar characteristics (e.g., schizophrenia, and unipolar depression)^16^ and antipsychotic medications that are used to treat manic or psychotic symptoms^17,18^ can reduce colour discrimination^19–21^. In addition, medications for BPD may also play some role in development of CV disorders; although drugs such as lithium are considered to rarely produce such disorders^22^.

Considering the issue of similar characteristics in the physiopathology, there is a shortage of studies that evaluate CV processing in BPD is observed. Two studies evaluated colour blindness in BPD^23,24^, but these studies presented conflicting results, since the former^23^ reinforces the hypothesis of X-linked dominant genetic transmission of affective liability, while the second^24^ states that bipolar and related illnesses are not transmitted by a single major gene close to the protan/deutan region of the human X-chromosome. Although relevant, these two studies involved heterogeneous samples, in addition to a high level of bias, both in relation to the low methodological control and in relation to the use of measurement instruments with little accuracy. Aligned with this, they only contemplate patients with colour blindness, which reduces the generalisation power of the results for other subpopulations of people with BPD.

Reliable tools are needed to evaluate acquired and congenital losses of colour discrimination^25,26^. Previous studies have employed diverse lighting conditions and different analytical procedures, the results of which have revealed small changes in CV but not sufficient changes to characterise the possible relationship between BPD and colour deficiency.

The Lanthony Desatured D15 (D-15d) test is commonly used to evaluate acquired and congenital defects in CV. Consisting of 15 caps, the chromatic saturation was reduced to two and the brightness increased to eight, being a modification of the classic D-15 test version^27^. The D15d is similar to the D-15 in administration but the colour samples are lighter and paler. With these modifications, the D15d test is more sensitive and widely used for early detection of CV losses in the early stages of deterioration^25^.

Unlike D-15 and D-15d tests^28^, there is less within-subject variability in test results for Cambridge Colour Test (CCT) subtests. The CCT allows precise control over chromaticity parameters of the stimuli, multiple randomised presentations of initial target–background chromaticity differences, and uses a staircase psychophysical procedure for estimating discrimination thresholds^29^. Thus, only one experimental session is generally sufficient to characterise colour detection thresholds, since repeated measurements are used per CCT subtests.

Two tests are available in the CCT: Trivector and Ellipse. The Trivector test is another way to assess congenital and acquired damage and can be performed quickly (i.e., <5 minutes). The advantage of computer control is that the difference between stimulus and background can be adjusted dynamically according to the individual performance^30^. The Trivector test uses pseudoisochromatic stimuli that exhibit dynamic variations in chromaticity between the targets and their background^31^. The Ellipse test uses described ellipses of discrimination (MacAdam ellipses) as parts of regions of the chromaticity diagram that contains all colors that are discriminated against all indiscriminate colors. MacAdam thus formed a measurement guide that indicates the accuracy of an individual’s color perception and whether it is impaired by anomalies that affect the nervous system^29,31^.

In view of these shortcomings in some previous studies of BPD, the objective of the present study was to determine whether visual colour processing is affected in BPD, and to investigate whether these deficits are associated with clinical and cognitive variables. Clinical features may interfere with the visual processing of psychiatric disorders^32^. Moreover, there is evidence that cognitive functioning is related to performance of psychophysical tasks^33,34^, so that cognitive processing could be partially motivated by perceptual deficits in BPD^35^. We hypothesised that there is an association between colour discrimination tasks and performance in cognitive tests. More specifically, our hypothesis is that deficiencies in the perceptual system could influence the performance of neuropsychological tasks, resulting in cognitive impairment. Finally, to ascertain the influence of factors such as age, gender and clinical characteristics, we will compare these variables between the control group and those affected by BPD.

## Results

The sample characteristics of the healthy control (HC) and BPD groups are summarised in Table 1. No significant differences between groups for age, level of education, or male:female ratio were found between groups.

**Table 1:** Sample characteristics of the HC and BPD groups (*n* = 24).

Variables	HC (*n* = 12)	BDP (*n* = 12)	*P-value*
**Gender**			
Male	7	7	*0.564* ^*a*^
Female	5	5	*0.564* ^*a*^
**Age**			
Age, year (*SD*)	33.6 (*6.0*)	32.3 (*5.2*)	*0.569* ^*b*^
**Level of Education**, **year (** ***SD*** **)**	8.9 (*1.2*)	8.8 (*2.1*)	*0.352* ^b^
**Age of onset**, **year (** ***SD*** **)**	—	21.1 (*1.4*)	0.001^b,*^
**Duration of illness, year (** ***SD*** **)**	—	11.3 (*4.9*)	*0.001* ^b,*^
**Number of hospitalizations**	—	1.8 (*0.7*)	*0.001* ^b,*^
**Young Mania Rating Scale (YMRS)**	—	20.2 (*4.3*)	*0.001* ^b,*^

^*^Statistically significant difference. ^a^ *χ* ^2^ test. ^b^Student’s *t*-test.

### Lanthony D-15d test

We calculated the Colour Confusion Index (CCI) for both groups. The CCIs in the BDP group were higher than the normative values that were reported in previous studies^25,36^. Descriptive statistics for the D-15d test are presented in Table 2.

**Table 2:** Descriptive statistics for CCI in the Lanthony D15d test (median, semi-interquartile range [sIQR], maxima and minima). For a perfect arrangement of colour caps CCI will be 1.0.

	Median	sIQR	Maxima	Minima
**HC**				
CCI	1.03	0.08	1.24	1.00
**BPD**				
CCI	1.71	0.27	3.08	1.31

**Figure 1:**
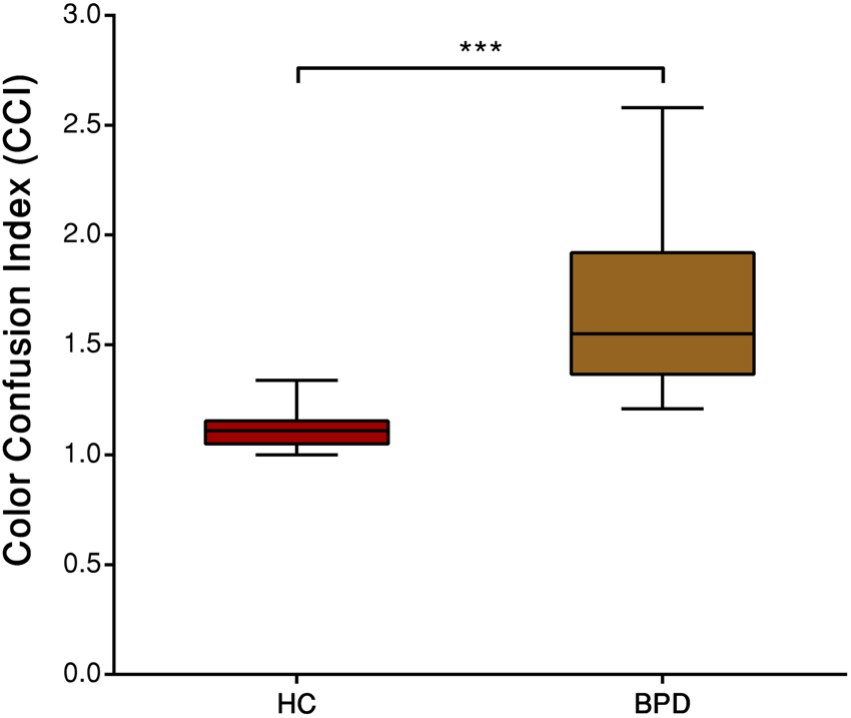
Boxplots of colour confusion indices in the healthy control (HC) and bipolar disorder (BPD) groups. ***p < 0.001.

The results of the D15d test are shown in Fig. 1. The Mann-Whitney *U* test indicated that CCIs medians in the BDP group were higher than in the HC group (*U* = 3, *r* = −0.81, *p* < 0.001).

Spearman’s rank correlation coefficient analysis did not indicate a significant association between CCIs and level of education (rho = −0.202, *p* = 0.344), gender (rho = −0.157, *p* = 0.508), or age (rho = −0.048, *p* = 0.824).

### Cambridge Colour Test

#### Trivector test

**Figure 2:**
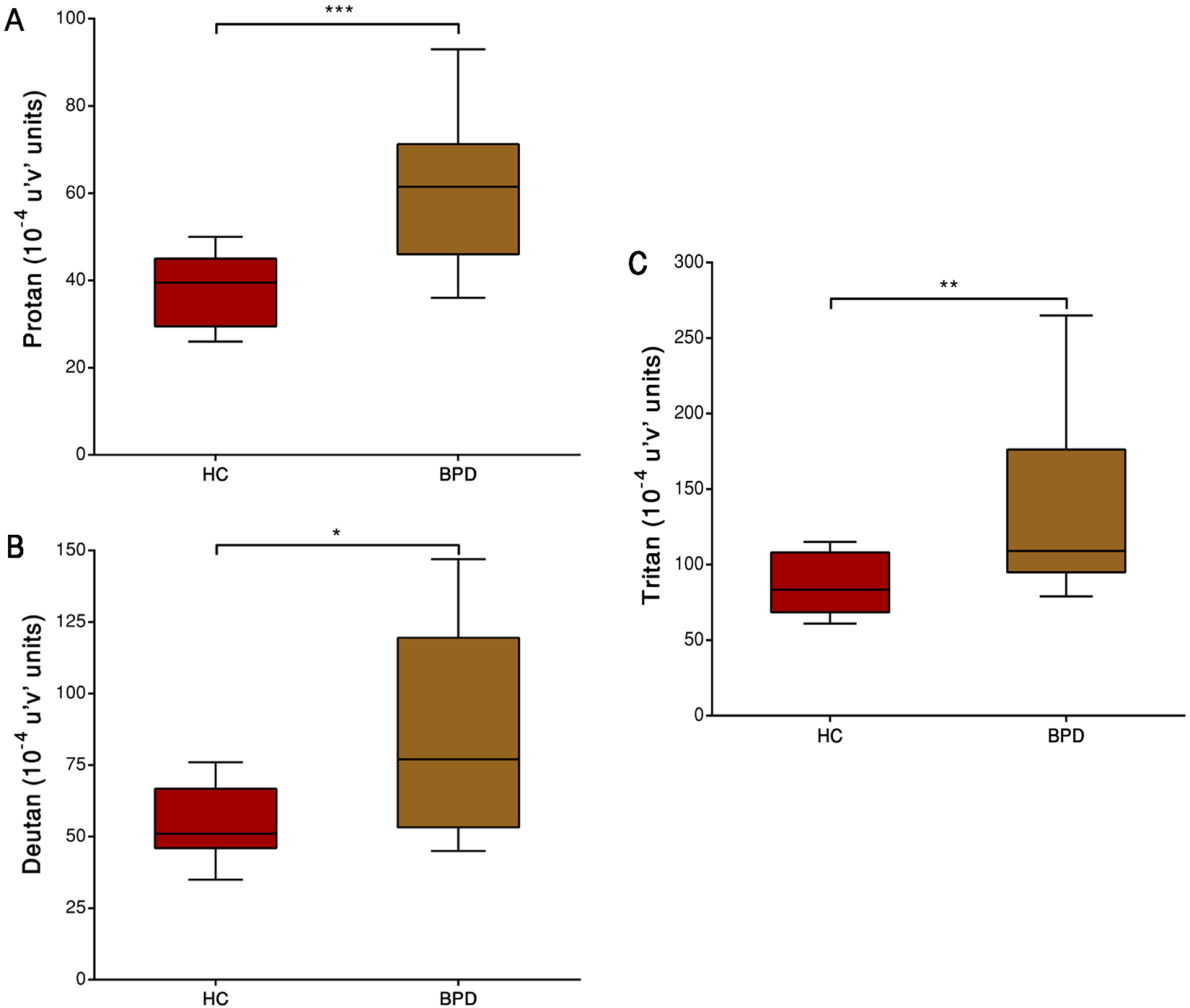
Trivector test, showing boxplots for protan (**A**), deutan (**B**), and tritan (**C**) confusion lines. The data are presented in 10-4 u’v’ units. Each boxplot is based on the results of 12 participants. *p < 0.05, **p < 0.01, ***p < 0.001.

Significant differences in discrimination thresholds along the three vector axes were found between groups. The results of the Trivector measurements are shown in Fig. 2.

Group comparisons revealed that the BPD group had higher chromatic discrimination thresholds than the HC group along the protan (*U* = 17, *r* = −0.65, *p* = 0.001), deutan (*U* = 31, *r* = −0.48, *p* = 0.021), and tritan (*U* = 20, *r* = −0.55, *p* = 0.007) axes. Spearman’s rank correlation coefficient analysis did not indicate significant associations between the trivector data and level of education (rho = −0.305, *p* = 0.248), gender (rho = 0.060, *p* = 0.589), or age (rho = 0.104, *p* = 0.599).

#### Ellipse test

**Figure 3:**
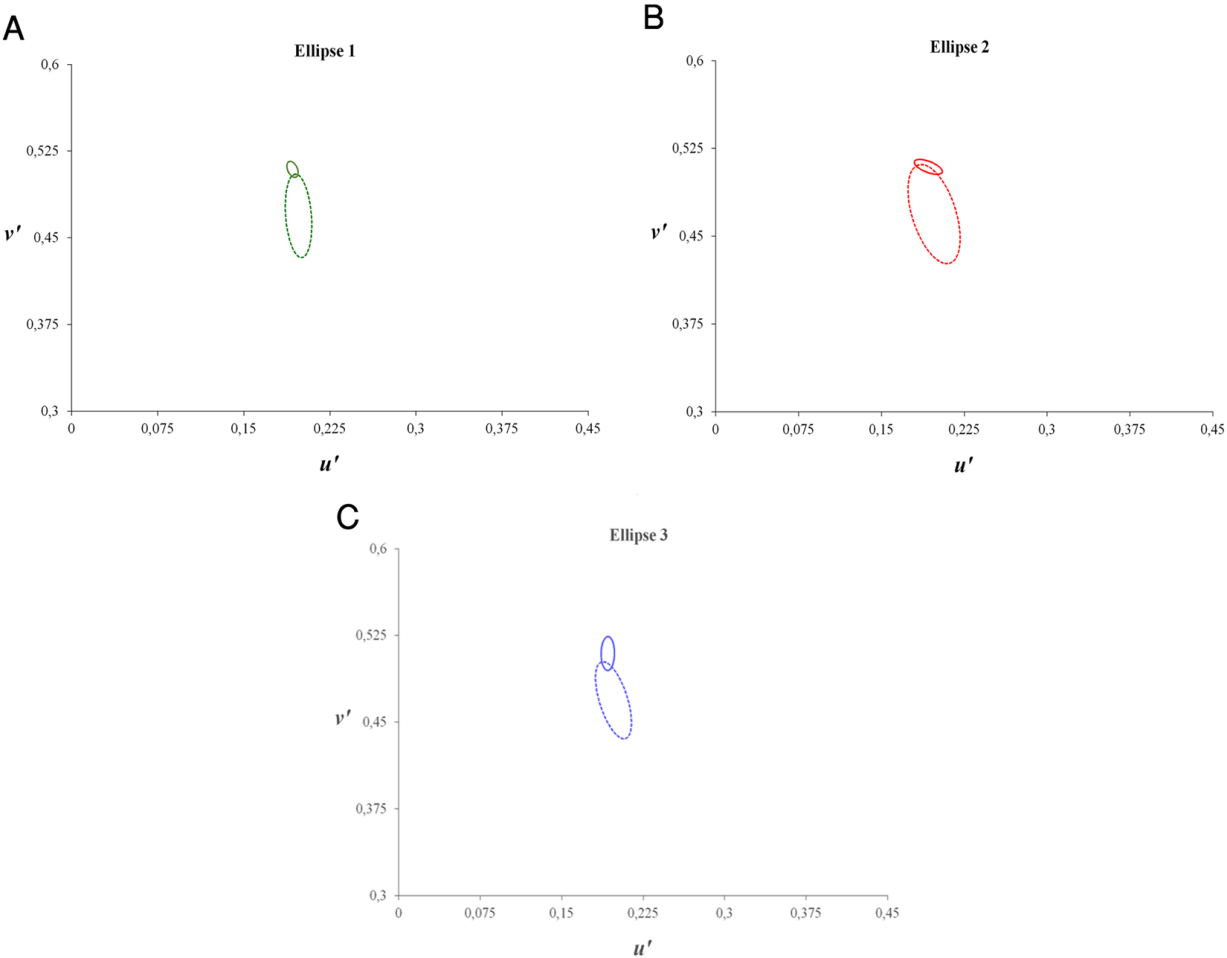
Mean colour discrimination ellipses. The data are plotted in the CIE 1976 Colour Space for 12 participants for Ellipse 1 (**A**), Ellipse 2 (**B**), and Ellipse 3 (**C**). Solid lines (for ellipses) represents healthy controls. Dashed lines (for ellipses) represent bipolar disorder patients.

The area of the ellipses represents colour discrimination. That is, the smaller the ellipse, the better the discrimination ability. Significant differences in discrimination thresholds along the three ellipse areas were found between groups. The results of the Ellipse measurements are shown in Fig. 3.

Group comparisons revealed that the BPD group had significantly larger ellipse areas than the HC group along the protan (*U* = 1, *r* = −0.84, *p* < 0.001), deutan (*U* = 6.5, *r* = −0.73, *p* < 0.001), and tritan (*U* = 21, *r* = − 0.60, *p* = 0.003) ellipses. Spearman’s rank correlation coefficient analysis did not indicate significant associations between the ellipse data and level of education (*p* > 0.05), gender (*p* > 0.05), or age (*p* > 0.05).

### Clinical measures and visual performance in BPD

The YMRS scale was positively correlated with colour discrimination tests (Table 3) among subjects with BPD, indicating that participants that presented more manic symptoms at the time of testing had worse visual processing performance. No significant correlations were found between scales that assess attention, memory, depression, sleep, or the tests involving CV.

**Table 3:** Correlation of visual tests and cognitive performance.

	Protan	Deutan	Tritan	CCI	Ellipse 1	Ellipse 2	Ellipse 3
YMRS	0.65^a^	0.37^a^	0.35^a^	0.67^a^	0.57^a^	0.38^a^	0.39^a^
Trail-Making	0.28	0.04	−0.06	0.22	0.10	−0.12	−0.18
MMSE	−0.17	−0.26	−0.31	−0.27	−0.06	−0.01	−0.01
Hamilton	0.04	0.15	−0.20	−0.06	0.20	0.13	0.16
Pittsburgh	0.06	−0.06	−0.06	0.22	0.13	0.54	0.60

^a^p < 0.01.

Linear regression analyses were conducted to determine the effect of cognitive performance on colour processing. It was observed that visual colour processing increased for CCIs and Ellipse Area 1 for each increase in the YMRS test scores (Table 4). Separate correlation analyses for each group showed that this relationship was found only for the BPD group (*p* < 0.05). No significant predictors for visual processing were found in relation to other cognitive tests (Trail-Making, MMSE, Flanker, Stroop, Hamilton or Pittsburgh).

**Table 4:** Linear regression analyses of the relationship between cognitive performance (YMRS) and visual tasks.

Dependent variable	Adjusted *R* ^*2*^ of model	β	*t*-value	*p*-value
Protan	0.36	0.43	1.23	0.23
Deutan	0.10	0.37	1.90	0.07
Tritan	0.08	0.35	1.78	0.08
CCI	0.43	0.67	4.33	0.01^a^
Ellipse Area 1	0.30	0.57	0.32	0.01^a^
Ellipse Area 2	0.11	0.38	1.96	0.06
Ellipse Area 3	0.12	0.39	2.03	0.05

^a^p < 0.05.

## Discussion

The main purpose of the present study was to investigate CV in patients with BPD as against HCs. We assessed CV under equivalent experimental conditions. Increases in thresholds (that is, lower colour discrimination) in the D-15d test (Fig. 1), Trivector test (Fig. 2) and Ellipse test (Fig. 3) were found in the BPD group. A lower threshold was associated with better colour discrimination. The results indicated that BPD patients had lower colour discrimination than HCs. For example, thresholds were ~1.5-times higher in the BPD group than in the HC group for CCIs and 1.5-, 1.4-, and 1.5-times higher in the BPD group than in the HC group for the protan, deutan, and tritan vectors, respectively. The average Ellipse area were two times larger in the BPD group. These results suggest the possibility that BPD patients may have impairments in the red-green and blue-yellow CV systems.

The visual performance was correlated with the degree of mania of the patients, since the linear regression indicated that YMRS was a significant predictor of visual performance. This relationship could be suggest differentially affected information processing streams in BPD^35^. In our study, the pattern of association between visual processing tasks and cognitive performance may be the result of neural reconnection during episodes of mania. This could be lead to increased vulnerability to stress and higher order cognitive dysfunction. These findings are consistent with reports of schizophrenia, indicating that lower level visual impairment may affect higher order cognition in ways that are distinguishable from HCs^37,38^. However, further studies are needed to establish causal inferences between visual impairments and possible consequences on the cognitive performance in those with BPD. However, we should mention that visual processing is composed in different stages. At early stages of the visual processing, some phenomena such as segregation of figure from background (as observed in the CCT test) and the detection of basic features like colour and stimulus orientation occur. This kind of processing seems not to be influenced by our knowledge, since they are largely automatic and independent of the cognitive domains^39^. To detect the stimulus, participants need to identify the orientation of the gap; thus, the identification of this pattern may require attention, mainly selective attention. Linear regression analysis for attention and inhibitory control tests (Stroop and Flanker) did not show any relation to performance in the visual task. The commonalities between perception and cognition are widely discussed by several authors^3,39,40^. In recent years, experiments involving neuroimaging has shown that attention increases the fMRI responses in visual cortical areas in a retinotopically specific manner both for endogenous and exogenous attention^41,42^. However, the data from this study are preliminary and do not ensure that cognitive capacity plays no role in visual tasks.

Furthermore, the use of a psychophysical method that has been widely used over the years tries to eliminate the relation between the participant’s sensitivity and possible bias^43,44^. Thus, the use of the tests we employed (mainly CCT subtests), under these conditions, sought to reduce the impact of inter- and intra-individual differences.

Regarding colour processing, trichromatic CV is based on the integration of the S, M, and L wavelengths by cone receptors. One consideration is the opponent process that results from excitatory and inhibitory connections between cones (red-green and blue-yellow CV systems)^45^. Both theories seek to explain optical and neural aspects of CV processing^3^. Our findings suggest that one or more of these CV systems may have been dysfunctional because we observed diffuse impairments for CCIs and also CCT Trivector and Ellipse. The use of these measurements may suggest the existence of possible visual pathways impairments^43,46^. Nonetheless, we cannot make definitive conclusions based only on the use of visual psychophysics, although these measures are known to be very reliable and can detect even subtle losses in CV. However, we can trace physiological parallels when we consider previous studies that used visual psychophysics to evaluate impairments under diverse conditions that affect the nervous system^47–50^.

Functional imaging studies have reported cortical thinning in BPD, indicating changes in blood flow and metabolism^51,52^. Evidence suggests the presence of pathophysiology in the ventral and orbital sectors of the prefrontal cortex in BPD. When we also consider changes that likely occur in the amygdala, we can speculate that both cortical and subcortical pathways are affected^53^. Ventral visual areas that are involved in the analysis of colour respond to cone activation, specifically S-cones^54^. Thus, any thinning or impairment in these areas may directly affect chromatic vision. Colour vision impairment in BPD may be related to regional cortical thinning in the left middle occipital cortex^15^, interactions between glia and neurons, and microglial overactivation^12^. Cortical thickness may affect primary visual cortex (V1) processing^55^. For example, cortical atrophy lead to changes in structure of early visual processing areas^55,56^ and colour processing is mediated by this pathway.

Interestingly, mood stabilizers in BPD have been shown to have beneficial effects in terms of some cognitive function in BPD patients compared with unmedicated patients^57,58^. Our BPD sample was composed of patients with type 1 BPD. The use of mood stabilizers, which reduce dopamine reuptake^59^, may be responsible for some differences in thresholds that were found between the BPD group and HC group. Such a possibility is supported by studies that investigated the effects of atypical antipsychotics, associated with the use of mood stabilizer, which enhanced visual sensitivity in schizophrenia patients^47,60^. However, in the present study, we did not subdivide our sample into mood stabilizer and mood stabilizer/antipsychotic subgroups because the sample sizes would have been too small. This should be investigated in future studies.

Consistent with the findings of O’Bryan *et al*. (2014), the present study provided new evidence of potential links between type 1 BPD and visual processing impairments. However, we assessed BPD and CV processing, whereas O’Bryan *et al*. (2014) investigated spatiotemporal processing. We hypothesize the existence of impairments in CV processing that may involve S, M, and L wavelengths. We observed significant changes in the red-green and blue-yellow CV systems^48^. The human visual system involves a combination of additive colour mixtures (L + M, L − M), and S − (L + M), and segregating the signals of different visual pathways can be difficult. Unclear are the ways in which the parvo-, magno-, and koniocellular pathways are affected by impairments to sensitivity to these wavelengths^61,62^. Such impairments may involve one or more of these pathways.

To our knowledge, no other studies have evaluated CV in BPD, thus demonstrating the novelty of our findings. Nonetheless, the present study has limitations. First, we conducted a behavioural study and did not investigate physiological links between BPD and CV. Second, even using procedures, including the D-15d, Trivector, and Ellipse tests, we did not account for all-important aspects of CV in BPD, relating, for example, short-term or long-term during pharmacological effects. Third, samples were relatively small. Finally, mood stabilizers and antipsychotics may have directly influenced CV impairments. We strongly recommend that future studies should combine multiple methods (e.g., imaging) with psychophysics to elucidate the mechanisms that are involved in CV impairments in type 1 BPD patients. In addition, although our results have demonstrated that patients with type 1 BPD had higher thresholds than HCs, we cannot ensure that BPD is a determining condition for impairment in colour vision. The scope of this study was not to establish a cause-and-effect relationship. The mechanisms that trigger chromatic losses may arise at the retinal level or reflect reduced cortical integration^63,64^ and this study is not able to provide a definitive basis for establishing the origin of colour vision loss in type 1 BPD. However, we must mention this study used a systematic methodology to evaluate colour discrimination thresholds for the groups through the application of standardized tests, with adequate levels of sensitivity and specificity and employed in previous studies involving colour vision^10,29,65,66^. Considering the homogeneity of our sample, the results indicated that the differences found are due to the independent variable; that is, the BPD clinical condition was associated with greater difficulty in discriminating colour when compared to participants without this condition. We reinforce that our results should be tested in controlled studies with larger samples, so they can be generalised to the BPD population more broadly.

In summary, our results showed that the differences in colour discrimination are marked in patients with BPD, which justifies further investigations in the search for pathophysiological mechanisms involved in sensorial alterations, in terms of colour discrimination threshold, in the set of clinical characteristics of BPD. This research suggests a new direction for studies and the need for research in this field of study.

## Methods

### Participants

Twelve HCs (*M* = 33.6 years, *SD* = 6.0 years) and 12 patients with type 1 BPD (*M* = 32.3 years, *SD* = 5.2 years), 25–42 years old, participated in the study. The BPD group consisted of outpatients (Illness duration: *Mdn* = 10.5 years; sIQR = 5.0 years) who were recruited from the Psychosocial Care Centre and were diagnosed with type 1 BPD by the institution’s psychiatrists based on the Structured Clinical Interview for DSM-5^67^. We also evaluated the patients using the Young Mania Rating Scale (YMRS)^68^. All of the patients met the criteria for acute mania (YMRS scores > 15)^69^ and were free of systemic conditions. The BPD patients were taking lithium (*n* = 10) or olanzapine (*n* = 2) for more than five years at the time of the study. The outpatients were taking only these medications with similar dosages. The participants in the HC group did not meet the criteria for specific Axis I or Axis II disorder according to DSM-5^70^.

The exclusion criteria were the following: (*i*) >45 years of age (since aging could affect the results^71^), (*ii*) history of neurological or cardiovascular disease, (*iii*) history of head trauma, (*iv*) history of chronic contact with substances such as organic solvents, and (*v*) current or prior drug abuse and the use of medications that may affect visual processing (except for the BDP group). The subjects were also required to have no ocular diseases, based on a fundoscopic exam and optical coherence tomography examinations. All of the participants were additionally screened for colour blindness using Ishihara’s test for colour deficiency^72^ and had normal or corrected-to-normal vision as determined by visual acuity of at least 20/20.

### Visual Measurements

#### Lanthony D15d test

The D-15d test is an arrangement test that is composed of an anchor reference cap and 15 other caps with different shades of the same value (8) and chroma (2) covered by Munsell papers^7,27^. The test was performed under standardised lighting conditions with a daylight fluorescent lamp that provided a colour temperature of 5000 K and 800 lux on the work plane. The bottom of the caps contained numbers from 1 to 15 that represented the correct sequence. The participants’ arrangement of the 15 caps was used to differentiate normal colour perception from moderate to strong congenital or acquired defects in deutan (green and green weak blindness), protan (red blindness), or tritan (blue-yellow blindness) colour discrimination. The results can be evaluated both qualitatively and quantitatively. The D-15d test results should not be analysed only qualitatively when early losses in CV need to be determined^73,74^. Thus, the Bowman’s CCI was used herein to quantitatively evaluate the performance of each participant^75^. A perfect arrangement of colour caps gives a CCI of 1, and indicates the severity of the CV deficiency. Normative CCI values of healthy individuals range from 1.00 to 1.30^25^.

#### Cambridge Colour Test

Stimuli were presented on a 19-inch LG cathode ray tube monitor (1024 × 786 resolution, 100 Hz refresh rate). The stimuli were generated using a VSG 2/5 video card (Cambridge Research Systems, Rochester, Kent, UK), which was run on a Precision T3500 computer with a W3530 graphics card. All of the procedures were performed in a room at 26 °C ± 1 °C. The walls of the room were covered in grey to better control luminance during the experiments. All of the measurements were performed with binocular vision. Monitor luminance was set, and chromatic calibrations were performed with a ColourCAL MKII photometer (Cambridge Research Systems).

The CCT is used to assess CV deficiencies as a rapid means of screening congenital or acquired deficits^30^. The CCT uses pseudoisochromatic stimuli (Landolt C) that are defined by the test colours that are to be discriminated on an achromatic background. The stimulus and background are composed of grouped circles with no spatial structure, the diameters of which are randomly varied (there is a 5.7° arcmin variation for the external diameter and 2.8° arcmin variation for the internal diameter). The procedure minimizes learning effects and guessing. These characteristics ensure that the participant can detect the target only by true CV and cannot use edge artifacts or luminance differences to answer. Thus, the present test does not require multiple applications, or that valuable time be spent in a preliminary equation of luminance test for each participant. The subject’s task being to press one of four corresponding keys (left, right, bottom or up). This task is cognitively simple and is readily grasped by subjects. The test should be conducted in a darkened room.

Both Trivector and Ellipse tests estimates sensitivity to S, M, and L wavelengths through the protan, deutan, and tritan confusion lines, respectively^30,76^. The staircases corresponding to the confusion lines are randomly intervealed, and occasional control trials are introduced to ensure the participant is alert and is not guessing^30^. The advantage of this brief test is that it can be performed in approximately five minutes and provides reliable results^10,26,29,77^. The three confusion axes converge at a co-punctal point (0.14744, 0.4184). The following u’v’ coordinates (CIE 1976) used were: protan (0.6579, 0.5013), deutan (−1.2174, 0.7826), and tritan (0.2573, 0.0000; for more detail, see^30^). The Ellipse test proceeds by a procedure on a specified number of radial vectors about a specified field point. The test searches two vectors at a time attempting to find the limit of the subjects colour discrimination along that vector. The parameters used by the CCT to form the ellipses are angle, axis ratio and ellipse length. The area of the ellipses represents colour discrimination in the u’v’units. That is, the smaller the ellipse, the better the discrimination ability. We used ellipse area to quantify possible losses in colour discrimination

We generally used a default setting in which the Landolt “C” had an opening at 1° of visual angle, minimum luminance of 8 cd/m², maximum luminance of 18 cd/m², 6 s response time for each trial, and distance of 269 cm between the participant and the computer monitor.

### Neuropsychological tests

#### Young Mania Rating Scale (YMRS)

Is a 11-item instrument used to assess the severity of mania in clinical trials of bipolar disorder^68^. The scores classify the type of mania observed. Mania: score greater than 19. Hypomania: score greater than 11, but less than 20. The higher the score, the greater the severity of the mania.

#### Trail Making Test (TMT)

This test was used to observe cognitive operations such as visual search, psychomotor speed, cognitive flexibility and sustained attention^78^. The individual was presented a sheet of randomly placed circles and was instructed to draw a line connecting the numbers (or numbers and letters) in corrects ascending sequence. A maximum time limit of 300 s was adopted. The measure used was the scoring errors for the whole test. The lower the number of errors, the better the participant’s performance.

#### Stroop Color-Word Interference

This test was used to measure executive function such as attention, cognitive flexibility, inhibition and information processing speed^79^. A series of colour words was presented to the participant and their task was to name the colour of each word presented. We used four colours (red, blue, yellow and green) in several combinations randomly displayed on a computer screen. The measure was a number of elements properly named. The smaller the number of errors in the incongruence of the stimuli, the better the performance.

#### Flanker Task

This task was used to evaluate attentional control and inhibition^80^. In the task, the stimuli (letters such as ZXYQ) were centrally presented when flanked by peripheral stimuli. We used the reaction time as a measure of cognitive ability. The lower the reaction time, the better the performance of the participant.

#### Mini-Mental State Examination (MMSE)

This test was used as a screening for possible cognitive impairment^81^. MMSE facilitates the detection of changes in mental states, as it allows the observation of verbal learning and memory, verbal and spatial working memory and semantic memory. The maximum score is 30. A score below 25 suggest possible impairment.

#### Hamilton Depression Rating Scale (HDRS)

Is a 17-item used to evaluate a possible depressive state in the participants^82^. Scores > 24 mean severe depression, > than 17 mean mild depression and < than 7 indicate absence of depression. HDRS is a short, reliable and easy-to-apply test.

#### Pittsburgh Sleep Quality Index

Is a 19-item self-rated questionnaire used to check for sleep quality among participants^83^. Losses in sleep could indicate reduction in attention and quality of life. The 19 questions are combined into seven components, each ranging from 0 to 3. Scores range from 0 to 21, with higher scores indicating poor sleep quality.

### Procedure

The procedures were performed in two stages. In the first stage, the participants were referred to our laboratory where we conducted the cognitive tasks. A specialist performed the neuropsychological tests. This procedure was performed in a quiet, comfortable, and reserved room; the approximate time was 1:30 h for each participant. In a second meeting, the participants performed the colour visual measurements. Each session of the second stage lasted from 45 minutes to 1 hour. Regarding all of these procedures, the participants were encouraged to take breaks between each block of measurements to avoid fatigue.

Prior to the start of the tests, instructions on the operation and tasks that individuals should perform were provided. Regarding the CCT, the tests only started when the individual understood that he should respond according to the opening of Landolt’s ‘C’. Regarding the D-15d test, it was explained the participants should organize the caps according to the nearest chromaticity.

#### Lanthony D-15d test

We applied the test binocularly. All sources of illumination beyond those of the test were excluded. The caps were randomised. The participants had to choose the cap that most closely approximated the colour of the anchor cap as they perceived it. Participants who have colour perception deficiencies have difficulty arranging the coloured caps and usually make mistakes^22^. In general, the participant’s task constituted to arrange caps according to order of greatest similarity to each preceding cap. The test was performed three times, using the mean of the applications, as the manual suggests^27,28^.

#### Trivector test

The four-alternative forced-choice (4-AFC)^9,29,44^ method was used. The subjects were instructed to indicate, using a remote control response box, the position of the opening/gap in the Landolt C stimulus. The participants were instructed to respond even if they could not identify the stimulus gap^30^. After each correct response, the chromaticity of the target proceeded closer to the chromacity of the background. Each wrong response or omission was followed by the presentation of the target at a greater chromatic distance from the background. The staircase step was doubled or divided by two after each incorrect or correct response, respectively. This procedure occurred throughout the experiment. The experiment ended after 11 reversals for each axis. The threshold per axis was estimated from the six final reversals^26,30^.

#### Ellipse test

Using the same procedure as the Trivector, the 4-AFC method was used. The ellipse testing protocol consisted of three MacAdam ellipses in the CIE (1976) u’v’ chromaticity diagram^30,84^. The discrimination threshold that was used in the present study was estimated from ellipses with eight vectors that were separated by 45° each and based on the adaptive staircase method. For every six consecutive reversions or errors per vector, the thresholds were formed to generate the ellipse, using the method of least squares^76^. The u’v’ coordinates were the following: Ellipse 1 (0.1977, 0.4689), Ellipse 2 (0.1925, 0.5092), and Ellipse 3 (0.2044, 0.4160). The ellipse colours were the following: Ellipse 1 (green), Ellipse 2 (red), and Ellipse 3 (blue). We used the area of ellipse as a measure.

### Statistical analysis

The statistical analysis was performed using SPSS 23.0 software. The distributions were assessed for normality using the Shapiro-Wilk test. The data from both groups presented a non-normal distribution; thus, nonparametric statistical tests were used to analyse the data. For all of the intergroup comparisons, the Mann-Whitney *U* test was used.

Linear regression analyses were carried out to evaluate the relationship between clinical measures, cognitive performance and visual colour processing between the groups. Regarding the clinical variables, scaled scores for YMRS were entered in the first block; Trail-Making, Flanker and Stroop in the second block; and Mini-Mental State Examination (MMSE), Hamilton Rating Scale for Depression, and Pittsburgh Sleep Quality Index in the third block, using the Enter method.

Pearson’s correlation coefficients (r) were used to assess the relationship between these clinical/cognitive variables and visual performance for each diagnostic group separately. Spearman’s rank correlation coefficients (rho) were used to assess relationships between the biosociodemographic variables (e.g., age, gender, years of education) and visual performance on the D-15d, Trivector and Ellipse tests. The effect size (*r*) was estimated based on z-score conversion^85^. Effect sizes > 0.50 were considered large effect sizes. The data in boxplots are presented with medians. In the boxplots, centre lines show the medians. Box limits indicate the 25th and 75th percentiles (determined by SPSS software). Whiskers extend 1.5-times the interquartile range from the 25th and 75th percentiles. The ends of the whiskers are the maximum and minimum values. Values of *p* < 0.025 were considered statistically significant.

### Ethics Statement

The present study was conducted in accordance with the ethical principles of the Declaration of Helsinki. The experiment protocols were approved by the Committee of Ethics in Research of the Health Sciences Center of Federal University da Paraiba (CAAE: 58677116.7.0000.5188). Written informed consent was obtained from all of the participants. All of the experiments were performed in accordance with relevant guidelines and regulations.
